# Advancing the United Nations Sustainable Development Goals Through Digital Health Research: 25 Years of Contributions From the Journal of Medical Internet Research

**DOI:** 10.2196/60025

**Published:** 2024-11-04

**Authors:** Raghu Raman, Monica Singhania, Prema Nedungadi

**Affiliations:** 1 Amrita School of Business Amrita School of Computing, Amrita Vishwa Vidyapeetham Kerala India; 2 Faculty of Management Studies University of Delhi New Delhi India

**Keywords:** sustainable development goal, topic modeling, public health, surveillance, gender equality, non-communicable disease, social media, COVID-19, SARS-CoV-2, coronavirus, machine learning, artificial intelligence, AI, digital health

## Introduction

Celebrating 25 years of the *Journal of Medical Internet Research*’s influence, we examine its contributions to the United Nations (UN) sustainable development goals (SDGs) [1]. Our study explores the interconnectedness of the SDGs using the SDG framework [2]. We aimed to:

- Analyze *Journal of Medical Internet Research* publications using a topic modeling approach to identify predominant topics;
- Map these topics to relevant SDGs to illustrate the journal’s impact on global health and equity;
- Demonstrate the interdisciplinary nature of digital health research and its relevance to sustainable development.

## Methods

Following the PRISMA (Preferred Reporting Items for Systematic Reviews and Meta-Analyses) guidelines, we conducted a comprehensive search on Scopus for articles published between 1999 and 2023 and found 8124 publications. Using inbuilt SDG mapping queries, we narrowed the results to 3550 publications (Multimedia Appendix 1) to identify relevant topics and their alignment with the SDGs [3]. The analysis used BERTopic, an advanced topic modeling technique, to review 25 years of *Journal of Medical Internet Research* publications. We used co-citation mapping to visualize SDG linkages and BERTopic to identify semantic connections [4].

## Results

**Figure 1:**
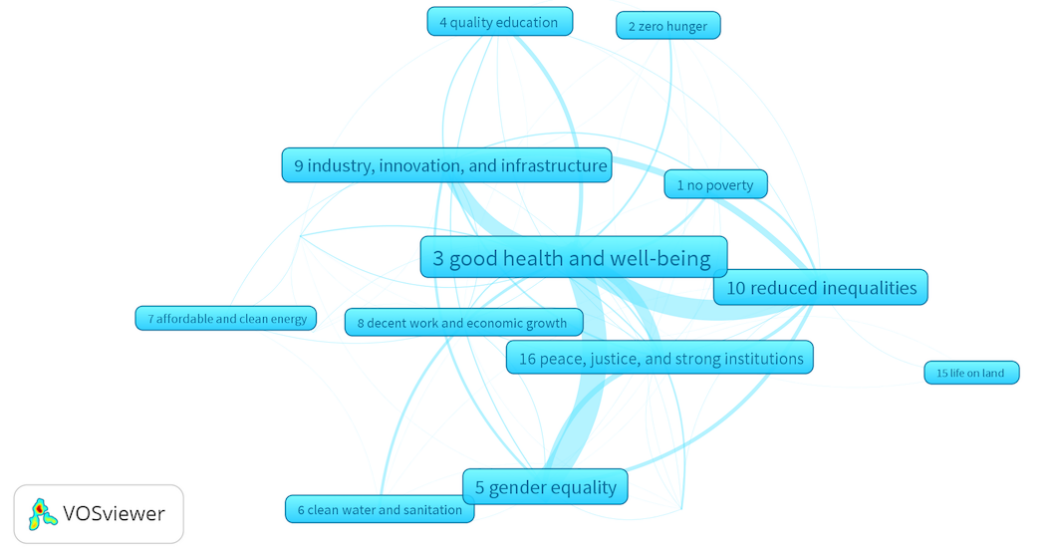
Interdisciplinary research published in the *Journal of Medical Internet Research* as a network of the United Nations sustainable development goals.

Figure 1 presents a network visualization of SDG interconnectivity to highlight the interdisciplinary nature of the research published in the *Journal of Medical Internet Research* [5]. Central to this network is SDG 3 (Good Health and Well-Being), which shows strong ties to SDGs 5 (Gender Equality), 10 (Reduced Inequalities), 16 (Peace, Justice, and Strong Institutions), and 9 (Industry, Innovation, and Infrastructure). These linkages highlight how advancements in health contribute broadly to gender equality, social equity, justice, and economic development, indicating a holistic approach that integrates health with key SDGs.

**Figure 2:**
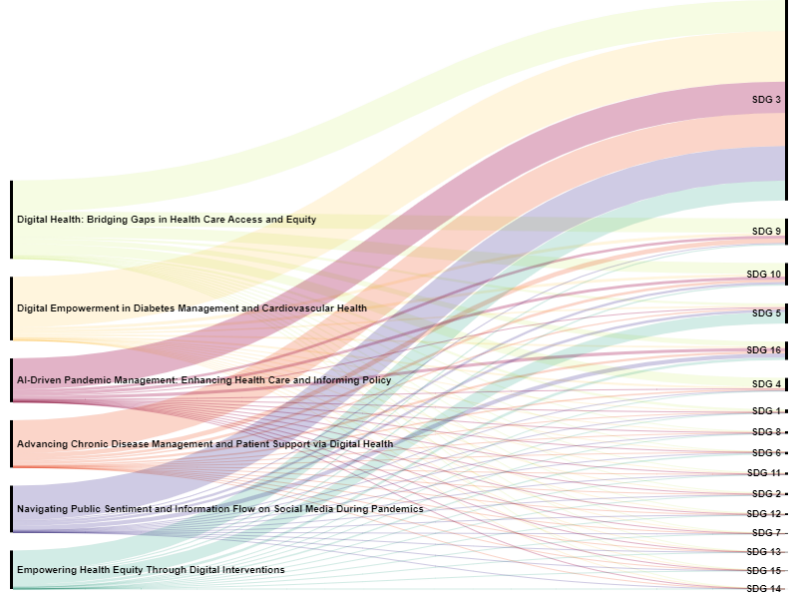
Major topics of works published in the *Journal of Medical Internet Research* mapped to the United Nations SDGs. AI: artificial intelligence; SDG: sustainable development goal.

Figure 2 shows major topics mapped to SDGs. SDG 3, which accounts for 79.8% of the publications, indicates that the journal primarily focused on health and well-being. Other notably featured SDGs include 9 and 10, with significant contributions to innovation in health care and reducing inequalities. SDGs 5 and 16 are also highlighted, supporting gender equality and the development of peaceful societies. Less-represented SDGs, such as SDG 4 (Quality Education) and SGD 6 (Clean Water and Sanitation), suggest expanding research areas, with environmental sustainability goals showing potential for future focus.

## Discussion

### Principal Findings

The 6 topics with significant keywords in the *Journal of Medical Internet Research* collectively emphasize the use of digital health to address diverse global health challenges, aligning with several SDGs (Multimedia Appendix 2). The significance of these topics is emphasized in studies such as those on chronic disease management [6,7], artificial intelligence (AI)–driven pandemic management [8,9], health care access [10], diabetes and cardiovascular health management [11], social media impact during pandemics [12], and health equity through digital interventions [13,14]. The topics below outline the diverse ways research from this journal has contributed to the SDGs:

- Advancing chronic disease management and patient support via digital health:
  Highlights how digital interventions support patients with chronic disease management from diagnosis to recovery, enhancing autonomy and treatment adherence
  Aligns with SDG 3
- AI-driven pandemic management:
  Showcases the use of AI in enhancing health care delivery and informing public health policies during pandemics
  Aligns with SDGs 3 and 9
- Digital health to bridge gaps in health care access and equity:
  Use of digital health technologies to make health care more accessible and equitable
  Supports SDGs 10 and 16 by improving access to quality care for underserved populations
- Empowering health equity through digital interventions
  Centers on reducing health disparities through digital interventions (eg, HIV prevention and health care for marginalized populations)
  Supports SDG 3.3 in combating communicable diseases
- Digital empowerment in diabetes management and cardiovascular health:
  Uses digital platforms to improve self-care, clinical outcomes, and health literacy in managing diabetes and cardiovascular diseases
  Aligns with SDG 3.4 in reducing mortality from noncommunicable diseases
- Navigating public sentiment and information flow on social media during pandemics:
  Explores how social media can shape public sentiment and spread information during health crises, including how misinformation affects public behavior and governance

Digitization can negatively impact the SDGs. COVID-19 worsened digital inequity, deepening divides in racialized communities and limiting access to essential services [15]. Significant challenges and financial losses in web-based research with underserved populations exist, emphasizing digitalization’s negative impact on data quality and resource efficiency [16]. To enhance the contributions of the *Journal of Medical Internet Research* to SDG 4 (Quality Education) and SDG 6 (Clean Water and Sanitation), we recommend prioritizing interdisciplinary research, launching special issues on these topics, forming partnerships with educational and environmental organizations, promoting specific funding opportunities, and organizing workshops.

### Limitations and Conclusion

Our study is constrained by the selected database and SDG mapping approach, which may limit its comprehensiveness. Additionally, while BERTopic modeling offers robust analysis, its inherent limitations and the subjective nature of topic interpretation could lead to oversimplifications and biases, necessitating expert review and validation. Despite these limitations, this study underscores the pivotal role of the *Journal of Medical Internet Research* in advancing the UN SDGs.

## Appendix

Multimedia Appendix 1 Dataset generated and analyzed during this study.

Multimedia Appendix 2 Major topics, top keywords, and SDG focus. AI: artificial intelligence; SDG: sustainable development goal.

## Abbreviations

AI: artificial intelligence
PRISMA: Preferred Reporting Items for Systematic Reviews and Meta-Analyses
SDG: sustainable development goal
UN: United Nations

## Data Availability

The dataset generated and analyzed during this study is available in Multimedia Appendix 1.
