# Supplementary figures and images for: Advancing the United Nations Sustainable Development Goals Through Digital Health Research: 25 Years of Contributions From the Journal of Medical Internet Research
Source: J Med Internet Res. 2024 Nov 4;26:e60025. doi: 10.2196/60025 (PMC11574500; doi:10.2196/60025)

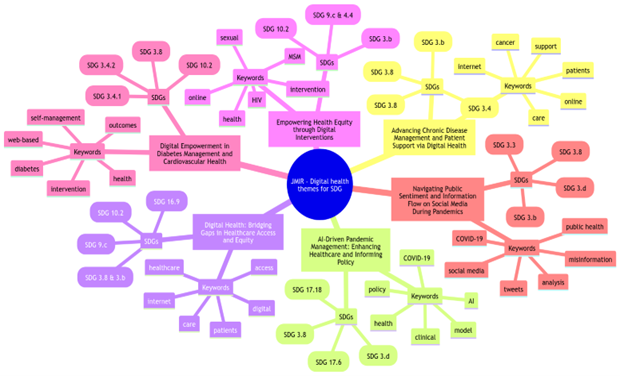

Supplement: Multimedia Appendix 2 [file jmir_v26i1e60025_app2.png]
